# Fenestropathy of Voltage-Gated Sodium Channels

**DOI:** 10.3389/fphar.2022.842645

**Published:** 2022-02-11

**Authors:** Tamer M. Gamal El-Din, Michael J. Lenaeus

**Affiliations:** ^1^ Department of Pharmacology, University of Washington, Seattle, WA, United States; ^2^ Division of General Internal Medicine, Department of Medicine, University of Washington, Seattle, WA, United States

**Keywords:** fenestrations, voltage-gated sodium channels, arrhythmia, Nav1.5, channelopathy, fenestropathy, LQT3 syndrome, Brugada syndrome

## Abstract

Voltage-gated sodium channels (Na_v_) are responsible for the initiation and propagation of action potentials in excitable cells. From pain to heartbeat, these integral membrane proteins are the ignition stations for every sensation and action in human bodies. They are large (>200 kDa, 24 transmembrane helices) multi-domain proteins that couple changes in membrane voltage to the gating cycle of the sodium-selective pore. Na_v_ mutations lead to a multitude of diseases - including chronic pain, cardiac arrhythmia, muscle illnesses, and seizure disorders - and a wide variety of currently used therapeutics block Na_v._ Despite this, the mechanisms of action of Na_v_ blocking drugs are only modestly understood at this time and many questions remain to be answered regarding their state- and voltage-dependence, as well as the role of the hydrophobic membrane access pathways, or fenestrations, in drug ingress or egress. Na_v_ fenestrations, which are pathways that connect the plasma membrane to the central cavity in the pore domain, were discovered through functional studies more than 40 years ago and once thought to be simple pathways. A variety of recent genetic, structural, and pharmacological data, however, shows that these fenestrations are actually key functional regions of Na_v_ that modulate drug binding, lipid binding, and influence gating behaviors. We discovered that some of the disease mutations that cause arrhythmias alter amino acid residues that line the fenestrations of Nav1.5. This indicates that fenestrations may play a critical role in channel’s gating, and that individual genetic variation may also influence drug access through the fenestrations for resting/inactivated state block. In this review, we will discuss the channelopathies associated with these fenestrations, which we collectively name “Fenestropathy,” and how changes in the fenestrations associated with the opening of the intracellular gate could modulate the state-dependent ingress and egress of drugs binding in the central cavity of voltage gated sodium channels.

## Introduction

**FIGURE 1:**
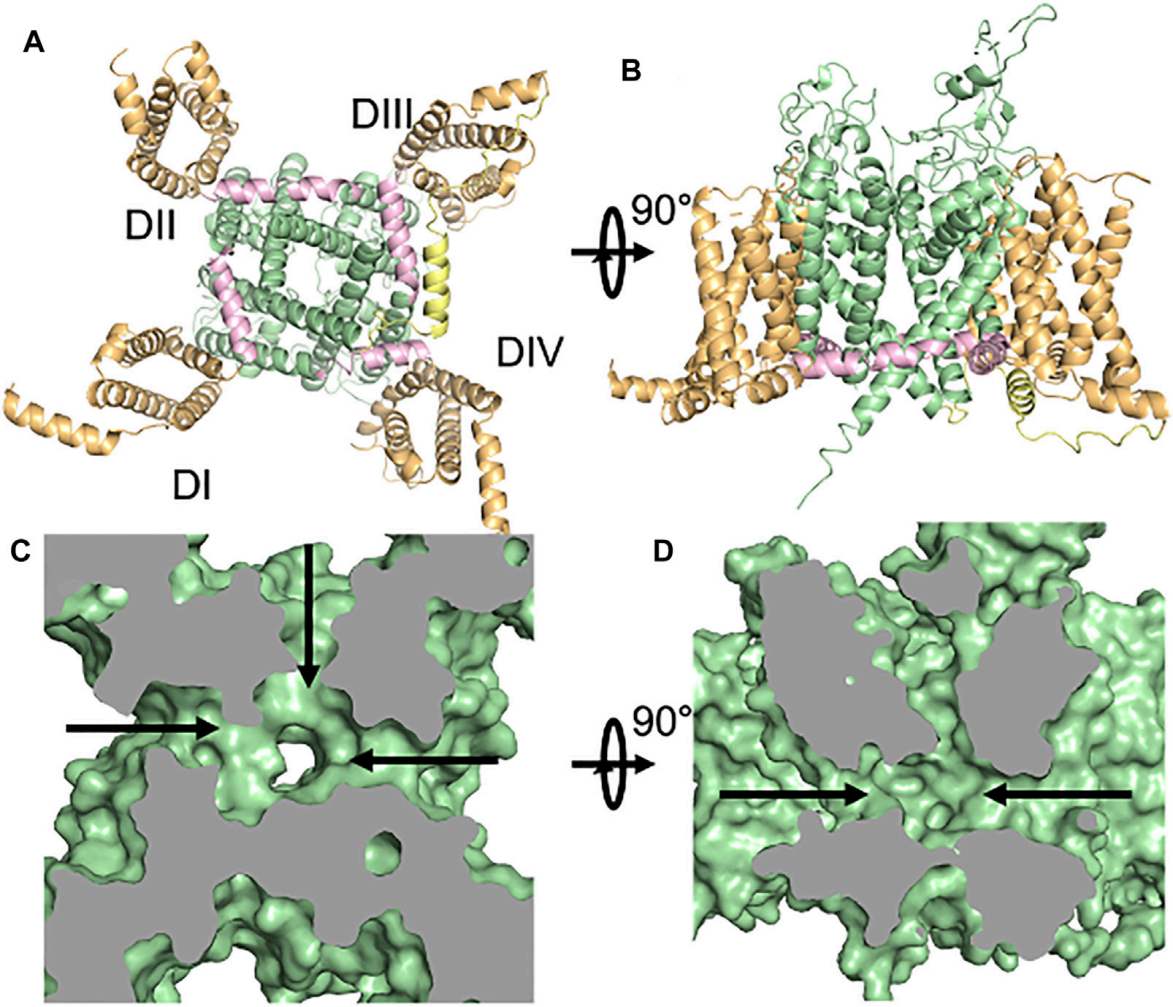
The structure of Na_v_1.5. **(A,B)** Orthogonal views of the cardiac sodium channel Na_v_1.5 with functional regions highlighted - voltage-sensing domain (orange), pore domain (green), S4-S5 Linker (pink), inactivation particle (yellow). **(C,D)**. Fenestrations in the pore-domain of Na_v_1.5 as shown in surface representations of the Cryo-EM structure. Orthogonal views of the protein’s van der Waals surface are shown with views as in **(A,B)** (Jiang et al., 2020).

**FIGURE 2:**
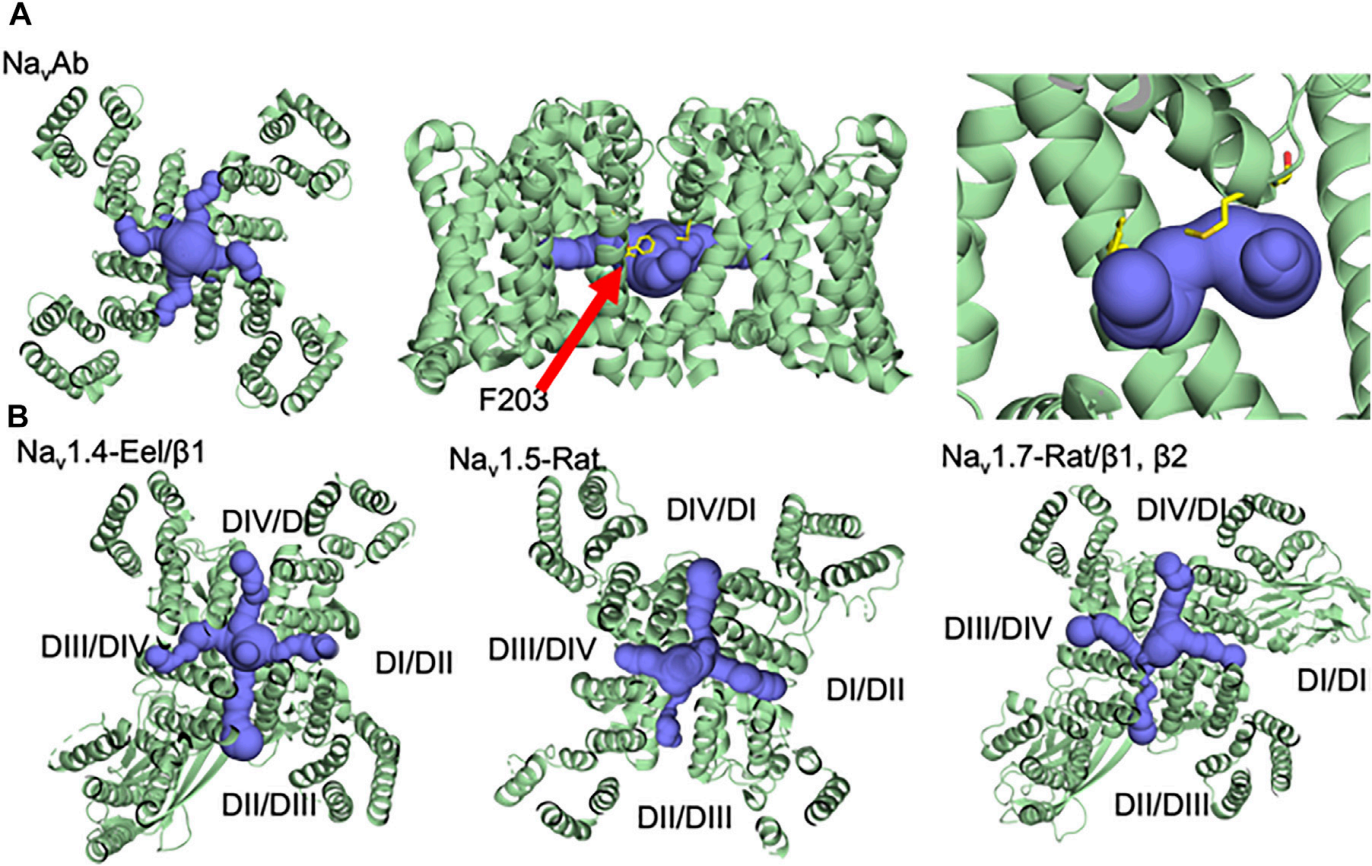
Fenestrations with different sizes and architecture in a variety of voltage-gated sodium channels **(A)** Na_v_Ab, **(B)** Na_v_1.4, Na_v_1.5, and Na_v_1.7 (Pan et al., 2018; Xu et al., 2019; Jiang et al., 2020).

Voltage-gated sodium channels (Na_v_) are the main triggers and propagators of action potentials (Catterall et al., 2020). These proteins were discovered in the 1950s by Hodgkin and Huxley and have since been studied by a variety of techniques, including cloning, overexpression/purification, electrophysiology, pharmacology, and structural biology. These studies have revealed the modular nature of the Na_v_ protein with each channel consisting of a voltage-sensing domain (VSD), linker region (S4–S5 linker), inactivation particle (IFM), and sodium-selective pore domain (PD) that work in concert to couple changes in membrane voltage to changes in membrane flux of sodium (Figures 1A,B). Nine different isoforms (Na_v_1.1–Na_v_1.9) have been discovered so far. Missense mutations in any of these regions of the nine different isoforms of Na_v_ lead to alteration of the way bioelectricity is initiated and conducted in any excitable organ, including heart and brain (Huang et al., 2017). Advances in sequencing technology, have recently shown that some of the missense mutations that lead to pathogenic phenotypes are part of an often-overlooked region of the channel, namely its “fenestrations” (Payandeh et al., 2011) (Figures 1C,D). These hydrophobic pathways, which connect the lipid phase of the plasma membrane to the hydrophilic central cavity, are not known to be important in voltage-sensing, gating, or coupling and their role in normal channel function remains unknown, though they have been shown to play a role in drug binding to the PD (Hille, 1977; Gamal El-Din et al., 2018b). Over billions of years of evolution, fenestrations have been conserved structures from prokaryotic to eukaryotic sodium channels (Gamal El-Din et al., 2018a). Structures of different Na_v_ have shown that the size of the fenestrations change during the gating cycle and that channels seem to have fenestrations of different sizes, perhaps reflecting a breadth of functions (Figure 2). Designing new drugs that aim to stabilize a specific functional state or bind to the central cavity should take these qualities into account. In addition to the previous points, we also discovered that some of the Na_v_ channelopathy mutations target fenestrations. In this review, we will shed light on the group of missense mutations that we collectively call “Fenestropathy” that lead to a change of the functionality of channel.

## Voltage-Gated Sodium Channels

Voltage-gated ion channels are transmembrane proteins that provide a hydrophilic pathway for ions, which cannot pass through the plasma membrane due to their charge. The voltage-gated sodium channel, Na_v,_ is composed of a complex containing a pore-forming α-subunit and up to two β-subunits, (Catterall et al., 2020). Nine different isoforms of Na_v_ α-subunits (Na_v_1.1–Na_v_1.9) and four sodium channel β-subunits have been discovered and functionally studied so far. The central nervous system (CNS) expresses four main isoforms (Na_v_1.1, Na_v_1.2, Na_v_1.3, and Na_v_1.6), while the peripheral nervous system (PNS) has three different isoforms (Na_v_1.7, Na_v_1.8, and Na_v_1.9) that are mainly responsible for nociceptive pain transmission. In skeletal muscles, Na_v_1.4 is the principal Na_V_, while Na_v_1.5 is the primary Na_V_ in cardiac muscle. The α-subunit is sufficient to form a functional Na_v_ while the β-subunits modulate the kinetics and voltage dependence of α-subunit activation and inactivation. The α-subunit is composed of ∼2,000 amino acid residues that form four homologous domains (I–IV), each with six transmembrane helices (S1–S6). Segments (S1–S4) form the voltage-sensing domain (VSD) (Figure 1A). The fourth transmembrane helix (S4) in each domain acts as the main voltage sensor with its characteristic positive residues at every third position. These amino acids (R or K) sense changes in the membrane’s electric field and transduce it to the pore domain (PD) formed by transmembrane helices S5 and S6. To gate the channel open, 6–14 elementary charges must move through the focused electric field around the VSD (Gamal El-Din et al., 2008; Hirschberg et al., 1995). Eukaryotic Na_v_ are predisposed to a wide range posttranslational modifications that change its expression and function. These include, but not limited to, phosphorylation, glycosylation, palmitoylation, ubiquitination, and methylation (Pei et al., 2018). The advancement of CryoEM technology enabled us to visualize a wide variety of eukaryotic Na_V_ structures, starting from cockroach (Na_v_Pas) (Shen et al., 2017), electric eel (Na_v_1.4) (Yan et al., 2017), rat heart (Na_v_1.5 in apo, drug-bound, and mutant forms) (Jiang et al., 2021; Jiang et al., 2020), to human brain (Na_v_1.2), muscle (Na_v_1.4) (Pan et al., 2018), heart (Na_v_1.5) (Li Z. et al., 2021), and nerve (Na_v_1.7) (Shen et al., 2019). All of these structures show the conservation of eukaryotic Na_V_ architecture from arthropods to humans. It reveals that Na_V_ contain four peripheral VSD (helices S1–S4) that are connected to the central, sodium-selective PD (helices S5–S6) by an amphipathic helix known as the S4–S5 linker (Figures 1A,B). These structures also provided some insights into the gating cycle of Na_v_ by showing the position of the inactivation particle (IFM) and the conformation of the open activation gate, by way of an inactivation deficient mutant of Na_v_1.5 (QQQ) (Jiang et al., 2021). Overall, these structures, in combination with functional data from similar constructs, highlighted the importance of distinct functional regions of the channel shown in Figure 1, including the VSD, PD, Elbow, S4–S5 Linker, IFM inactivation particle, activation gate, and selectivity filter. These structures also revealed the presence of hydrophobic pathways, or “fenestrations” between the transmembrane helices of the PD’s subunits (Figures 1C,D).

In contrast to eukaryotic Na_V_ channels, bacterial Na_V_ channels are composed of four identical subunits of ∼270 residues. Each subunit has a VSD and a PD (Payandeh et al., 2011) like their eukaryotic peers. They share the major biophysical features with their eukaryotic counterparts (Ren et al., 2001; Gamal El-Din et al., 2013; Gamal El-Din et al., 2019), though lack auxiliary subunits and posttranslational modifications. Crystallization of full-length bacterial Na_V_ channels like Na_v_Ab (Payandeh et al., 2011), Na_v_Rh (Zhang et al., 2012), and Na_v_Ms (McCusker et al., 2012) and analysis of their structures at high resolution have made them invaluable models for studying the structural basis of ion conduction, activation, inactivation, and drug interaction. They have also provided the only model to date of fenestrations and their potential roles in gating and drug binding (Lenaeus et al., 2017; Gamal El-Din et al., 2018b).

## Structure-Function of Fenestrations

### Fenestrations Before the Structure Era

In 1977, Hille hypothesized the existence of fenestrations by showing that the hydrophobic form of local anesthetics (LA) could reach their binding site even when the internal mouth of the sodium channel was closed (Hille, 1977). The existence of a hydrophobic entrance pathway distinct from the permeation pathway was very intriguing and has been borne out repeatedly by functional experiments from different labs (Bean et al., 1983). Over decades of electrophysiology and mutagenesis it has been established that there are three kinds of interaction of LA and antiarrhythmic drugs (AAD) with Na_V_: 1) resting state binding, which happens through the fenestrations, 2) use-dependent block, which occurs between open and resting states, and before entering the slow inactivated state, where the open activation gate is the main entrance pathway, and 3) inactivated state binding, where fenestrations role as an access pathway comes back. The binding affinity rank between these states differs for each Na_V_ blocker, though it is most commonly, Inactivated > Open > Resting and state-dependent binding is key to the mechanism of action of most Na_V_ blockers.

### Fenestrations as Drug Access Pathway

Despite the long-standing evidence for the existence of hydrophobic fenestrations in Na_v_, they were not visualized until the publication of the crystal structure from the prokaryote *Arcobacter butzleri* (Na_v_Ab) in 2011 (Payandeh et al., 2011). As expected from years of functional studies, the Na_v_Ab structure showed that the fenestrations are the lipid exposed part of the pore domain connecting the central cavity of the channel to the hydrophobic lipidic portion of the membrane (Figure 2A). The size of the fenestrations in the first reported pre-open state of Na_v_Ab was ∼8 × 10 Å (Payandeh et al., 2011). Lipids were found to penetrate deeply into the central cavity through the fenestrations and block the ion conduction pathway. Other bacterial sodium channel structures of the *magnetococcus* bacterium (Na_v_Ms) and marine *alphaproteobacterium HIMB114* (Na_v_Rh), showed similar fenestrations, which suggested that these pathways were a conserved architectural feature of sodium channels (McCusker et al., 2012; Zhang et al., 2012). The first resolved structure of a eukaryotic sodium channels (Na_v_Pas) reveals four fenestrations, three closed and only one (DIII-DIV) is open (Shen et al., 2017). In contrast, the activated/inactivated structure of the electric eel Na_v_1.4 channels shows four open side-portals (Figure 2B left). These fenestrations were varied in their size and shape, with the biggest one being between domains III and IV, perhaps indicating the role played by this specific fenestration to act as an access pathway for drugs in the resting and inactivated states (Yan et al., 2017). The structure of the cardiac sodium channel Nav1.5 also showed different fenestration sizes; DII-DIII fenestration is the biggest one and DIII-DIV is much smaller (Jiang et al., 2020) (Figure 2B middle). The slow inactivated structure of Na_v_Ab and the structure of Na_v_1.7 provided additional insights into the dynamicity of fenestration’s structure. In case of Na_v_Ab, fenestration’s size could change with the slight turning of one phenylalanine ring (F203), while the rotation of two phenylalanine rings between domains I and IV, in case of Nav1.7, closes the hydrophobic side portal (Payandeh et al., 2012; Shen et al., 2019) (Figure 2B right). The finding that the size of the fenestrations changes during the gating cycle, from resting to activated/inactivated, and that channels seem to have fenestrations of different sizes/shapes, perhaps reflects the diversity of its function.

### The Role of Fenestrations in Pharmacology

**FIGURE 3:**
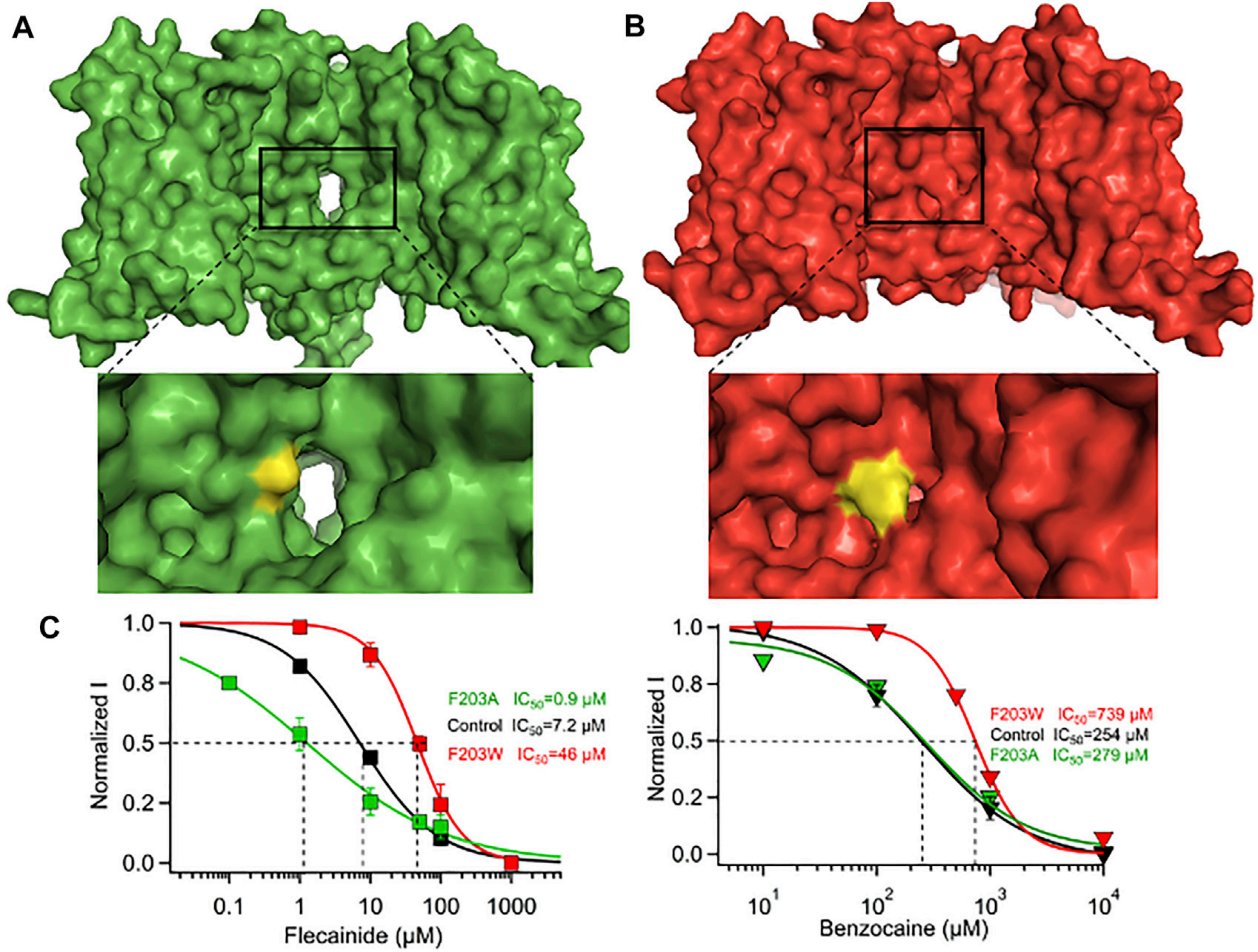
Effect of F203A/W mutations on the size and functionality of Na_v_Ab. **(A)** F203A mutation enlarges the fenestrations significantly and causes a significant leftward shift in the steady-state inactivation profile. **(B)** F203W mutation narrows the fenestrations significantly and causes a significant rightward shift in the steady-state inactivation profile. **(C)** Effect of F203A and F203W on the potency of flecainide (left) and benzocaine (right) (Gamal El-Din et al., 2018b).

It has been suggested that the DII-DIII or DIII-DIV fenestrations are key pathways for drug access to the central cavity, though direct evidence for this is currently lacking in eukaryotic Na_V_. In Na_v_Ab, a conserved hydrophobic residue (F203) was shown to play a major role in drug binding by controlling access to the central cavity for drugs like flecainide (Gamal El-Din et al., 2018b) (Figure 2A middle). Mutating F203 to tryptophan occluded the fenestrations almost completely, while mutating it to alanine makes the fenestrations wider (Figures 3A,B). This change in fenestration’s size increased the potency of bulky drugs like flecainide by more than 50-fold between big fenestrations (A203) and small ones (W203) (Figure 3C, left). Small drugs like lidocaine and benzocaine showed a smaller change in potency between big and small fenestrations, showing the importance of matching the drug’s size to fenestration’s size. In addition to the gateway residue F203, four residues form the bottleneck part of the fenestrations in Na_v_Ab: M174, T175, T206, and M209 (Figure 2A). These amino acids form the main architecture of the fenestration’s gate. In Na_v_1.5 structure, the corresponding region of fenestrations between DI and DII is formed by residues N927, L404, and M923, while that in the fenestration between DIII and DIV is lined by I1756, I1757, F1760. The residues which form the entrance of the four fenestrations in Na_v_1.5 are shown in Table 1. These residues are conserved in the nine different isoforms of Na_v_ and it has been shown that mutation of the fenestration’s lining residues increases the resting-state affinity of some LA’s by 15-fold (Ragsdale et al., 1994). Mutation of I1756 to cysteine in hNa_v_1.5 has also been shown to create an alternative pathway that allows the rapid diffusion of flecainide out of Nav1.5 (Ramos and O'Leary M, 2004).

**TABLE 1:** Amino acids that form the fenestrations in hNav1.5.

DI S6: M394, I397, F398, S401, L404, N406, L407
DII S6: M923, V924, N927, N930, N933
DIII S6: I1454, L1462, N1463, I1466, I1469, N1472
DIV S6: I1756, I1757, F1760, V1763, M1766
P1-helix-SF-DI: F366, M369, T370
P1-helix-SF-DII: F892, L895, C896
P1-helix-SF-DIII: L1413, A1416, T1417, F1418
P1-helix-SF-DIV: F1705, T1708, T1709

### Fenestrations as Lipids Binding Sites

The structures of Na_v_Ab, Na_v_Ms, and Na_v_Rh and cryo-EM structures of multiple eukaryotic Na_v_ have revealed lipid molecules in their fenestrations. This conserved interaction between lipids and Na_v_ fenestrations over millions of years of evolution suggests that lipids are key to fenestrations, though that role is unclear. Molecular dynamics studies have suggested that fatty acids chains can reach the central cavity of Na_v_ through the fenestrations, perhaps suggesting a direct effect on drug ingress/egress (Kaczmarski and Corry, 2014). We observed the displacement of the acyl chains in many of our structures when we add a drug of interest to Na_V_, as have others. A recent study has also shown that poly unsaturated fatty acids (PUFA) can inhibit Nav1.5 and cause a leftward shift in the steady state inactivation profile. The authors of this study suggested that PUFA could permeate through DIII-DIV fenestrations and inhibit the channel, which would be consistent with the lipid positions in our structures and those of others. Another study showed that EPA (eicosapentaenoic acid) effectively inhibited Na_V_ in a dose-dependent manner and shifted the steady-state inactivation curve to the hyperpolarizing direction, which resulted in a reduced sodium window current (Liu et al., 2018). These studies suggest that fatty acids dock their hydrophobic chain in the fenestrations and let its charged head interact with sodium ions blocking its conduction through the pore (Bohannon et al., 2020).

### Genetics/Fenestropathy

**FIGURE 4:**
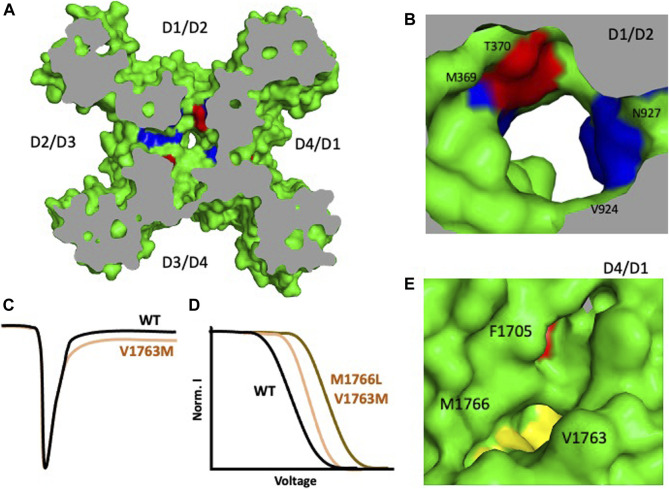
Some arrhythmia mutations in hNa_v_1.5 mapped to the channel’s fenestrations. **(A)** Overall structure of hNav1.5 shows the fenestropathy mutations. LQT3 mutations are shown in red, Brugada syndrome mutations in blue, and mutations which cause mixed phenotype are shown in yellow. **(B)** D1/D2 fenestration shows four residues that cause LQT3 and Brugada syndrome. **(C)** V1763M leads to late sodium current **(D)** M1766L causes a significant shift in the steady-state inactivation profile. **(E)** D4/D1 fenestration shows F1705S, V1763M, and M1766L (Li Z. et al., 2021).

Advances in gene sequencing have enabled the identification of many missense mutations which are linked to diseases, including Na_V_ channelopathies. More than 1,000 missense mutations have been linked to the nine isoforms of Na_v_ and almost half of these mutations target the human cardiac sodium channel Na_v_1.5 to cause arrhythmia syndrome and related disorders (Huang et al., 2017). Overall, mutations of SCN5A which encodes hNav1.5 account for approximately 5–10% of cases of LQTS (Tester et al., 2005). About 80% of the missense mutations in Nav1.5 lead to either Long QT syndrome (LQT3) or Brugada syndrome (BRGDA1). Most of these missense mutations are in the classical regions of the channel (including the VSD, S4–S5 linker, IFM, PD), though we also discovered some that occur in the four fenestrations of the channel (Tables 1, 2) (Figure 4) - which was surprising due to unknown function of these regions. Our previous work with the bacterial sodium channel Na_v_Ab about the effect of fenestration’s size on AAD potency showed us the effect of how fenestrations’ mutations could lead to a change in the biophysical properties of the channel (Gamal El-Din et al., 2018b). We found that F203W significantly shifted the steady state inactivation profile of the channel rightward compared to the WT. It also changed the kinetics of the channel’s inactivation during the pulse. In contrast, we found that F203A mutations shifts the steady-state inactivation curve leftward (Gamal El-Din et al., 2018b). These findings triggered our interest about the effect of fenestrations’ missense mutations on sodium channels functionality. When we switched to hNav1.5, we found that most of these mutations are in the DI-DII and DIV-DI fenestrations affecting mainly the entrance or the bottle neck area of the architecture of these side portals (Figure 4, Table 2). We also noticed that four of these mutations change the architecture of the roof of the fenestrations by changing some residues of the P1 helix. Interestingly, we observed that Brugada mutations are mainly concentrated around the P1 helix part of the fenestrations, while LQT3 are more localized at the sides or the bottom of the fenestrations. One example is the M1766L in DIV S6, which exists in the bottom side of DIV-DI fenestrations. This fenestration appears to be closed in rat Na_v_1.5 structure (Jiang et al., 2020) while it looks slightly open in hNa_v_1.5 (Li Z. et al., 2021). M1766L leads to LQT3 syndrome by causing a significant decrease in the sodium channel expression and a 10-fold increase in the persistent late sodium current compared to wild type channel (Valdivia et al., 2002). The second mutation V1763M also led to late sodium current and a significant rightward shift in the inactivation curve of hNa_v_1.5 channel (Chang et al., 2004). V1763 is a neighboring residue to M1766, and both seem to seal the DIV-DI fenestration. One way to think about why these mutations cause late sodium current and significant leftward shift of the steady state inactivation profile is that methionine is bulkier than valine and leucine and changing the size of the residues at this critical area may lead to a change in the movement of S6 during fast inactivation resulting in inefficient closing of the activation gates and the resulting effects (Figures 4C–E). Another example is found with the T1709M and T1709R mutations. Both lead to Brugada syndrome with a significant reduction of channel’s expression (Kapplinger et al., 2010). Another important mutation is F1705S which causes SIDS. This mutation causes a hyperpolarizing shift of the steady-state inactivation profile in addition to a delayed recovery from inactivation. These biophysical features would reduce the availability of Na channels and delay the conduction of cardiac impulses. Overall, these kinetic properties of F1705S could result in a decrease of net sodium current and loss of function feature that supports a proposed linkage between Brugada syndrome and SIDS (Otagiri et al., 2008). Most of the other mutations listed in Table 2 are associated with a clear phenotype but the biophysical characteristics of them are not studied yet.

**TABLE 2:** Missense mutations that target the fenestrations of hNa_v_1.5

Mutation	Isoform	Location	Pathology
M369K	hNav1.5	DI-P1 helix	Brugada
T370M	hNav1.5	DI-P1 helix	LQT3
I397T	hNav1.5	DI S6	LQT3
L404Q	hNav1.5	DI S6	LQT3
F892I	hNav1.5	DII-P1 helix	Brugada
C896S	hNav1.5	DII-P1 helix	Brugada
V924I	hNav1.5	DII S6	Brugada
N927S	hNav1.5	DII S6	Brugada
S1458Y	hNav1.5	DIII S6	LQT3
N1472S	hNav1.5	DIII S6	LQT3
F1705S	hNav1.5	DIV S5-S6	SIDS
T1709M/R	hNav1.5	DIV S5-S6	ICEGTC
V1763M	hNav1.5	DIV S6	LQT3
M1766L	hNav1.5	DIV S6	LQT3
Y1767C	hNav1.5	DIV S6	LQT3

Na_v_1.4 sodium channel is the main isoform expressed in skeletal muscles, where its main responsibility is to couple the action potential to the activation of voltage-gated calcium channels and the subsequent rush of Ca^+2^ to trigger muscle contraction. So far, seven main disorders result from the channelopathy of Na_v_1.4; Myotonia, para-myotonia congenita, hypokalemic periodic paralysis, hyperkalemic periodic paralysis, normokalemic periodic paralysis, congenital myasthenia, and congenital myopathy with hypotonia (Cannon and Bean, 2010). We found that some of the mutations that cause these diseases are in the fenestrations of Na_v_1.4. For example, M1592V, which causes hyperkalemic periodic paralysis, is in the fenestration between DIV and DI (Rojas et al., 1991). Another neighboring residue in the same fenestration, V1589 can cause para-myotonia congenita if it is mutated to methionine (Heine et al., 1993). These two residues V1589 and M1592 are the equivalent residues of V1763 and M1766 in Na_v_1.5.

The diseases caused by the missense mutations in the fenestrations of voltage-gated sodium channels highlight the importance this newly apparent functional region of Na_V_. We are uncertain of the route by which these mutations result in diseases, though speculate that it could be related to a change in the way that these residues interact with the activation/inactivation gates, or it could stem from the fact that changing the hydrophobicity of the fenestrations residues will lead to a change in the way lipid molecules interact with the fenestrations and thus the whole channel. Further experiments will need to be performed to determine the effects of these disease-associated mutants.

### Fenestrations in Other Ion Channels

Fenestrations have been identified in many other ion channels, including potassium channels, calcium channels, and others (Zhou et al., 2001; Miller and Long, 2012; Lenaeus et al., 2014; Dong et al., 2015; Wang and MacKinnon, 2017; Zhao et al., 2019). Fenestrations in these channels are frequently bound to lipids or detergents in structural experiments, but their native function and the reason for broad representation in ion channels remain poorly understood. These regions are present in the earliest structures of ion channels, though were largely overlooked until the term “fenestration” was coined with the discussion accompanying publication of Na_v_Ab’s structure in 2011 (Payandeh et al., 2011). Subsequent re-analysis of KcsA (the bacterial potassium channel from *Streptomyces lividans*) showed these regions offered state-dependent access to the central cavity of potassium channels, could act as state-dependent drug binding sites, and that their occupancy by lipids or drugs influenced the stability of the potassium channel selectivity filter (Zhou et al., 2001; Lenaeus et al., 2005; Lenaeus et al., 2014). The most clear-cut role for this region in gating comes from the dimeric, mechanosensitve two-pore (K2P) channels. K2P channels are leak potassium channels that regulate the resting membrane potential and a variety of structures have been solved from this family, including TRAAK, TREK, and others (Brohawn et al., 2012; Miller and Long, 2012). Data from these channels have revealed state dependent binding of alkyl chains to their inter-domain fenestrations and suggested a regulatory role for fenestration-bound lipids or detergents (Dong et al., 2015). A similar role for fenestration/lipid interactions have also been shown in transient receptor potential (TRP) channels responsible for pain and temperature sensation, amongst other functions (Lichtenegger et al., 2018).

Ion channel fenestrations have also been shown to act as drug binding sites in other ion channels. Norfluoxetine has been shown to bind to the fenestration of K2P channels and displace alkyl chains in a state-dependent manner (Dong et al., 2015). The selective anti-epilectic (AED) Z9444 was shown to bind in the fenestration between domains II and III in Ca_v_3.1 (Zhao et al., 2019), and a wide swath of ligands have been shown to bind to the fenestrations of the clinically important human ether-a-go-go related gene (hERG) potassium channel (Kamiya et al., 2006). The cryo-EM structure of the second-generation antihistamine drug astemizole bound to hERG has recently been published, confirming the hypothesis that compounds are stabilized in the hERG channel by a hydrophobic interaction in its fenestrations (Wang and MacKinnon, 2017; Asai et al., 2021).

Several newer studies also highlight the potential importance of these regions in future drug discovery, including a possible role for providing subtype specificity in combination with protein- or tissue-specific lipids. The fenestrations of the voltage-gated potassium channel KCNQ4 (Kv7.4), for example, have been shown to bind the activating AED retigabine when phosphatidylinositol 4,5 bisphosphate (PIP_2_) binds nearby (Li T. et al., 2021). K_v_7.1 has also been shown to have a novel, specific drug-binding site that is only available when the channel associates with a KCNE beta subunit, thereby highlighting one potential method of subtype-specific targeting in otherwise highly homologous ion channels (Wrobel et al., 2016).

## Conclusion

Put together, these findings highlight the importance of the fenestration region of Na_v_ and ion channels in general. Despite this, very little is known about their native function, their role in pathophysiology, as well as their role in drug binding *in vivo*. We think the term “Fenestropathy” helps group a variety of genetic and acquired disorders to this often-overlooked region of ion channels and propel research in this area.
